# Supplementary material for: A set of multi-entry identification keys to African frugivorous flies (Diptera, Tephritidae)
Source: Zookeys. 2014 Jul 24;(428):97–108. doi: 10.3897/zookeys.428.7366 (PMC4143993; doi:10.3897/zookeys.428.7366)
Supplement: Supplementary material 6 — Key to Ceratitis [file zookeys-428-097-s006.zip › SF6_ZooKeys_key to Ceratitis/key/SF6_ZooKeys_key to Ceratitis/Media/Html/Ceratitis flexuosa .htm]

Ceratitis (Pterandrus) flexuosa (Walker)


***Ceratitis*** **(*Pterandrus*) *flexuosa*
(Walker)**

*Trypeta flexuosus* Walker, 1853: 382.

Body
length: 4.52 (3.75-4.75) mm; wing length: 5.00 (3.85-5.45) mm.

Male

Head: Antenna yellowish orange. First flagellomere twice as
long as pedicel. Arista with short to moderately long rays; ventral rays
shorter and sparser than dorsal rays, especially basally. Frons convex, pale
yellow; with short scattered setulae largely of same color as frons. Frontal
setae well developed. Face yellowish white; gena somewhat darker. Genal seta
and setulae dark, well developed.

Thorax:
Postpronotal lobe yellowish white, without spot. Mesonotum: ground color pale
gray with faint orange tinge; microtrichose silvery shine; with streaks and
darker markings but without distinct spots, except at transverse suture and
around dorsocentral and (to lesser extent) prescutellar seta implant dark,
shiny spots, prescutellar yellowish white markings separate. Scapular setae
dark. Scutellum yellowish white, basally without spots, apically with three
strongly reduced dark spots. Subscutellum yellowish orange, without distinct
spots. Anepisternum on ventral half darker yellow but often not pronounced;
setulae pale.

Legs:
Yellow except where otherwise noted; setation typical for subgenus, mixed pale
and dark. Foreleg: femur without bushy pilosity, with dispersed dark setulae,
ventral setae black. Midleg: femur anteriorly with large median white spot;
along dorsal edge on distal half to 0.6 with short dense black pilosity,
ventrally with few long black setulae near base; tibia dilated, with apical
0.33 to 0.4 brown with silvery spot, only conspicuous when viewed from
certain angle; with black feathering dorsally and ventrally along distal 0.4 to
half; anteriorly with third row of very short black feathering, area between
third and dorsal row not dark. Hindleg: femur at apical 0.25 with longer
setulae dorsally and ventrally.

Wing:
bands largely reduced to isolated brownish spots, especially marginal band.
Medial band absent; crossvein R-M just proximal to middle of discal cell. Apex
of vein R_1_ distal to level of crossvein R-M. Crossvein DM-Cu oblique
posterobasally.

Abdomen:
Mostly yellowish orange. Tergites 2, 4 and 5 largely grayish (not silvery);
tergite 3 with posterior 0.33 brownish, interrupted medially; tergite 5 with
anterior part brownish or yellowish brown, usually as two separate spots,
occasionally as continuous band. Male epandrium in lateral view with posterior
lobe of lateral surstylus short and stout, slightly curved; anterior lobe well
pronounced.

Female

As
male except for the following characters: Darker patches on gena not distinct.
Subscutellum usually with obscured dark spot in the middle. Legs without
feathering; forefemur posteroventrally with pale pilosity. Abdominal tergite 5
anteriorly at most with darker yellow markings. Oviscape shorter than
preabdomen. Aculeus at most six times longer than wide; tip with distinct
apical indentation and lateral margin slightly sinuous. Some female specimens
have a generally darker appearance.

(Description
after De Meyer & Freidberg, 2006)
